# Characterization and Assessment of Native Lactic Acid Bacteria from Broiler Intestines for Potential Probiotic Properties

**DOI:** 10.3390/microorganisms12040749

**Published:** 2024-04-07

**Authors:** Xiaoxia Li, Wang Li, Longmei Zhao, Yuanxiao Li, Wanling He, Ke Ding, Pinghua Cao

**Affiliations:** College of Animal Science and Technology, Henan University of Science and Technology, Luoyang 471023, China

**Keywords:** probiotics, antimicrobial activity, cell surface characteristics, antibiotic susceptibility, poultry

## Abstract

Probiotics are the most promising alternative to antibiotics for improving animal production and controlling pathogenic infections, while strains derived from natural hosts are considered highly desirable due to their good adaptation to the gastrointestinal tract. The aim of this study was to screen *Lactobacillus* with broad-spectrum antibacterial activity from broilers fed an antibiotic-free diet and evaluate their potential as poultry probiotics. A total of 44 lactic acid bacteria (LAB) strains were isolated from the intestines of healthy broilers, among which 3 strains exhibited outstanding antimicrobial activity and were subsequently identified through 16S rRNA sequencing as *Enterococcus faecium* L8, *Lactiplantibacillus plantarum* L10, and *Limosilactobacillus reuteri* H11. These three isolates demonstrated potent bacteriostatic activity against *Staphylococcus aureus*, *Listeria monocytogenes*, *Escherichia coli*, and *Salmonella cholerae*, with inhibition zones ranging from 15.67 ± 1.53 to 21.33 ± 0.58 mm. The selected LAB strains exhibited high tolerance to acid and bile salts, with *L. reuteri* H11 displaying the highest survival rate (ranging from 34.68% to 110.28%) after exposure to 0.3% (*w*/*v*) bile salts for 6 h or a low pH environment (pH 2, 2.5, and 3) for 3 h. Notably, *L. reuteri* H11 outperformed other strains in terms of hydrophobicity (84.31%), auto-aggregation (53.12%), and co−aggregation with *E. coli* ATCC 25922 (36.81%) and *S. aureus* ATCC 6538 (40.20%). In addition, the three LAB isolates were either fully or moderately susceptible to the tested antibiotics, except for strain L8, which resisted gentamycin and vancomycin. Consequently, these three LAB strains, especially *L. reuteri* H11, isolated from the intestines of broiler chickens, represent promising probiotic candidates that can be employed as feed additives to enhance production performance and control poultry pathogens.

## 1. Introduction

In recent decades, the breeding industry has witnessed the excessive and irrational use of antibiotics, significantly contributing to the growing threat of bacterial antimicrobial resistance. This issue poses risks to both animal and human health [1]. Recognizing this challenge, the European Union took a significant step by prohibiting antibiotics in animal feed in 2006 [2]. Similarly, China’s Ministry of Agriculture and Rural Affairs, through Notice No. 194, enforced a ban on commercial feed containing growth-promoting drug additives (except for Chinese herbal medicines), effective from 1 July 2020 [3]. However, the use of subtherapeutic antibiotics in the breeding industry is still permitted in some countries, like China, to combat bacterial diseases, which can provoke greater bacterial resistance [4,5]. As a result, researchers have been exploring safe, cost-effective, and efficient alternatives to ensure the long-term sustainability of the livestock and poultry industry [3]. Unquestionably, probiotics continue to be the most promising natural candidates [5]. In response, there has been a surge of interest in employing probiotics as an alternative to antibiotics in livestock and poultry production for disease prevention and enhanced growth performance [6].

Probiotics are non-pathogenic living microorganisms that confer health benefits when administered in appropriate quantities [7]. They are primarily sourced from dairy products, food, fermented products, animal and human gastrointestinal tracts, and so on [5,6,8]. Lactic acid bacteria (LAB), encompassing various species such as *Enterococcus*, *Lactobacillus*, *Pediococcus*, *Streptococcus*, *Lactococcus*, *Vagococcus*, *Leuconostoc*, *Oenococcus*, *Weissella*, *Carnobacterium*, and *Tetragenococcus*, have been extensively researched as probiotics over the years [9]. Extensive studies have demonstrated that LAB can adhere to and thrive in the gastrointestinal tract of animals by competitively inhibiting pathogen binding and producing antimicrobial substances like organic acids, bacteriocins, and hydrogen peroxide, thereby playing a stabilizing and protective role within this ecosystem [10,11]. Furthermore, LAB can influence digestive and metabolic processes, regulate immune responses and cytokine production, and enhance the integrity of the intestinal mucosal barrier through competitive exclusion [11,12,13]. These attributes contribute to improved animal health, enhancing production performance [14] and bolstering host immunity [15]. Nevertheless, the effectiveness of probiotics is contingent on the specific species and strains used [11]. Therefore, any potential probiotic candidate must meet the criteria of safety (non-pathogenic and susceptibility to antimicrobials), in vivo benefits (lactic acid production and antagonism against pathogens), and resilience within the host’s gastrointestinal environment (tolerance to acidic pH and high bile salt concentrations) [16]. Furthermore, an ideal candidate must display effective adhesion to the intestinal epithelium and establish a symbiotic colonization with the natural gut microbiota [17]. Consequently, rigorous selection criteria must be implemented to assess the probiotic properties of candidate strains [18].

Despite the development of a substantial number of commercially available probiotic strains in recent decades, there remains a pressing need for superior strains compared to the existing ones [6]. Moreover, the viability and inconsistent results of probiotic strains in many cases hinder their widespread use [5]. By comparison, isolating probiotic strains from their natural hosts is highly desirable, as such native strains are already adapted to the gastrointestinal tract. Consequently, when administered to animals, they exhibit better adherence and survivability, enabling them to exert the expected beneficial effects more effectively than strains from other sources [19]. Thus, developing host-specific probiotic strains is imperative to maximize health benefits and enhance animal production performance [20]. Accordingly, we isolated LAB strains from the intestinal tracts of healthy broilers fed an antibiotic-free diet rich in prebiotics. We evaluated their probiotic characteristics in vitro and screened potential probiotic isolates for application in poultry farming.

## 2. Materials and Methods

### 2.1. Isolation of LAB Strains

LAB strains were isolated from healthy broilers on antibiotic-free chicken farms. Briefly, the birds were humanely euthanized by CO_2_ inhalation followed by cervical dislocation. The intestinal tracts were aseptically excised and promptly transported to the laboratory for microbial isolation. After removing the intestinal contents, the mucosa of each intestinal segment was meticulously scraped into 40 mL of sterile normal saline and homogenized for 30 min at 37 °C with continuous shaking. The samples were appropriately diluted, and 0.1 mL of the diluted suspension was evenly spread onto de Man, Rogosa, Sharpe (MRS) (AOBOX, Beijing, China) screening plates containing 0.004% (*w*/*v*) bromocresol purple (Sigma-Aldrich, St. Louis, MO, USA) and 2% (*w*/*v*) CaCO_3_ (Sigma-Aldrich, St. Louis, MO, USA). Subsequently, the plates were incubated for 48 h at 37 °C under anaerobic conditions. Only colonies displaying a yellow zone and a calcium-dissolving zone were selected (presumed to be LAB) and subjected to three successive transfers on MRS agar plates using the dilution-streaking method to obtain individual colonies.

The presumed LAB isolates were then subjected to further characterization, including cell morphology assessment, Gram staining, and catalase reaction. Isolates that were catalase-negative and Gram-positive were chosen and preserved at −80 °C in MRS broth with 20% glycerol (Sigma-Aldrich, St. Louis, MO, USA). Before each use, these isolates were resuscitated through sub-culturing in MRS broth.

### 2.2. Antimicrobial Activity

To evaluate the antimicrobial activity of the cell-free supernatant (CFS) of the selected LAB against pathogens, the Oxford cup agar diffusion method [21] was employed. In brief, the LAB isolates were cultured in MRS broth at 37 °C for 24 h, and the CFS was obtained by centrifugation at 5867× *g* for 10 min, followed by filtration sterilization using a sterilized 0.22 µm MILLEX^®^GP filter unit (PES membrane) (Merck Millipore, St. Louis, MO, USA). Subsequently, 200 μL of the overnight cultures of indicator pathogens (10^7^ CFU/mL) was evenly spread onto the surface of nutrient agar plates. Sterile Oxford cups (outer diameter of about 8.0 mm) (Shanghai Jinpan Biotech Co., Ltd., Shanghai, China) were placed on the inoculated plates and filled with 100 μL of LAB CFS. Following incubation at 37 °C for 18–24 h, the inhibition zones (including disc size) of the LAB were measured by using a pair of Vernier calipers. The indicator pathogens included *Staphylococcus aureus* ATCC 6538, *Listeria monocytogenes* ATCC 10403S, *Escherichia coli* ATCC 25922, and *Salmonella cholerae* ATCC 13312. LAB strains with noteworthy antimicrobial activity were selected based on comprehensive cluster analysis for further investigations.

### 2.3. Characterization of LAB Antimicrobial Compounds

The LAB isolates with higher antimicrobial activity were selected and tested to determine the nature of the produced antimicrobial substances—primarily organic acids, hydrogen peroxides, and bacteriocins. This characterization followed the method described by Reuben et al. [19] with some modifications. Specifically, the CFS of LAB, as prepared previously, was separated into five portions that underwent different treatments: one remained untreated as a control, the second was adjusted to a pH of 5.0 using NaOH, the third was treated with 1.5 mg/mL catalase (Sangon Biotech Co., Ltd., Shanghai, China) at 37 °C for 2 h, the fourth was treated with 1 mg/mL proteinase K (Sigma-Aldrich, St. Louis, MO, USA) at 37 °C for 2 h, and the fifth was heat-treated (boiled) for 20 min. Exactly 100 μL of CFS from each treatment was loaded into sterile Oxford cups placed on the agar plates previously inoculated with 200 μL of indicator pathogens (10^7^ CFU/mL). These indicator pathogens included *E*. *coli* ATCC 25922 (representative of Gram-negative bacteria [G^-^]) and *S*. *aureus* ATCC 6538 (representative of Gram-positive bacteria [G^+^]). The plates were then incubated at 37 °C for 18–24 h, and the inhibition zones (including disc size) of the LAB were measured by using a pair of Vernier calipers.

### 2.4. Acid and Bile Tolerance Tests

The acid tolerance of the LAB isolates was assessed following the method described by Wang et al. [22], with some modifications. Overnight LAB cultures were resuspended in MRS broth at pH levels of 2.0, 2.5, and 3.0 after centrifugation and then adjusted to a concentration of 10^7^ CFU/mL. The suspensions were subsequently incubated for 3 h at 37 °C, with samples collected at different intervals (0, 1, 2, and 3 h). These samples were serially diluted with PBS (Sangon Biotech Co., Ltd., Shanghai, China) and plated on MRS agar plates. After 48 h of incubation at 37 °C, the viable colonies were counted.

To evaluate bile salt tolerance, overnight LAB cultures were washed twice with sterile PBS at pH 7.2 after centrifugation. Subsequently, the cell pellets were resuspended in fresh MRS broth containing 0.3% and 0.5% bile salt (Solarbio, Beijing, China) and adjusted to a concentration of 10^7^ CFU/mL. The cell suspensions were then incubated for 3 h at 37 °C, with samples taken at two different intervals. The gradient-diluted samples were spread on MRS agar plates and incubated at 37 °C for 48 h, after which the viable cells were counted.

### 2.5. Cell Surface Characteristics

The cell surface characteristics of LAB isolates, including hydrophobicity, auto-aggregation, and co-aggregation with pathogens, were determined as previously described [22] with some modifications. The cell pellets of LAB and pathogens (*E. coli* ATCC 25922 and *S. aureus* ATCC 6538) were washed twice and resuspended with sterile PBS. The cell suspensions were adjusted to an optical density at 600 nm (OD_600_) of 0.5 ± 0.05 (*A_i_*).

#### 2.5.1. Auto-Aggregation

Then, 5 mL of LAB cell suspension was vortexed for 10 s and then incubated for 4 h at 37 °C. The absorbance of the supernatant was read at 600 nm (*A_t_*). The auto-aggregation rate was calculated as follows:

Auto-aggregation (%) = [1 − (*A_t_*/*A_i_*)] × 100

where *A_i_* was the initial OD_600_ of LAB suspension, and *A_t_* was OD_600_ after 4 h of incubation.

#### 2.5.2. Co-Aggregation

To assess co-aggregation, 2 mL of each LAB cell suspension (*A_s_*) was mixed with each pathogen suspension (*A_p_*) in equal volume, vortexed for 30 s, and subsequently incubated for 4 h at 37 °C. The absorbance of the supernatant of each mixed suspension was measured at 600 nm (*A_m_*). The co-aggregation rate was calculated according to the formula below:

Co-aggregation (%) = [1 − *A_m_*/(*A_s_* + *A_p_*)/2] × 100

where *A_s_* and *A_p_* were initial OD_600_ of LAB and pathogenic bacteria and *A_m_* was OD_600_ of the mixed suspension after 4 h of incubation.

#### 2.5.3. Cell Surface Hydrophobicity

Exactly 3 mL of LAB cell suspension was mixed with 1 mL of *o*-xylene (Sigma, USA) and then vortexed for 1 min. The mixture was stood at 37 °C for 20 min and separated into two phases. The aqueous phase was collected, and the OD_600_ was measured (*A_t_*). The cell surface hydrophobicity was calculated as follows:

Hydrophobicity (%) = (1 − *A_t_*/*A_i_*) × 100

where *A_i_* was the initial OD_600_ of LAB and *A_t_* was OD_600_ of the aqueous phase after 20 min of incubation.

### 2.6. Safety Evaluation in Vitro of LAB Strains

#### 2.6.1. Hemolytic Activity Assay

The LAB strains were inoculated on Columbia blood agar (Sangon Biotech Co., Ltd., Shanghai, China) and incubated for 24 h at 37 °C for hemolysis [23]. The hemolytic activity of the strain was shown by β-hemolysis (a clear, colorless/lightened yellow zone around the colony). In contrast, hemolytic inactivity was manifested as α-hemolysis (a zone of greenish to brownish discoloration surrounding the colony) and γ-hemolysis (no change).

#### 2.6.2. Antibiotic Susceptibility Test

The antibiotic susceptibilities of the LAB isolates were evaluated through the disc diffusion method [24]. To elaborate, the overnight cultures of the LAB strains designated for testing were adjusted to achieve a concentration of 10^7^ CFU/mL, and then they were evenly spread onto MRS agar plates. Subsequently, ten types of commercially available antibiotic discs (Hangzhou Microbial Reagent Co., Ltd., Hangzhou, China) were aseptically placed on the surface of the MRS agar. These antibiotics included chloramphenicol (30 μg), erythromycin (15 μg), tetracycline (30 μg), gentamicin (10 μg), ampicillin (10 μg), vancomycin (30 μg), streptomycin (10 μg), rifampicin (5 μg), kanamycin (30 μg), and novobiocin (30 μg). Following an incubation period of 24 h at 37 °C, the diameters (mm, including disc size) of the clear zones surrounding each antibiotic disc were measured by using a pair of Vernier calipers. The LAB isolates were classified as either resistant (≤15 mm), intermediately sensitive (16–20 mm), or sensitive (≥21 mm) in accordance with previously established criteria [25].

### 2.7. Molecular Identification of LAB Strains

The genomic DNA of the LAB isolates was extracted using a bacterial DNA extraction kit (Tiangen Biotech, Beijing, China) for molecular identification. The sequences of 16S rRNA were amplified using universal primers 27F and 1492R, as previously described [26]. The PCR products were sequenced by Sangon Biotech Co., Ltd. (Shanghai, China). The sequence data obtained were subjected to online homology analysis using NCBI BLASTn for the final identification of LAB.

### 2.8. Statistical Analysis

Statistical analyses were conducted using IBM SPSS Statistics version 27.0 software (IBM Corporation, Armonk, NY, USA). One-way ANOVA was employed to determine the significance of differences among the mean values. When necessary, Duncan’s multiple range test was used to identify differences between means. A significance level of *p* < 0.05 was considered statistically significant. All data were expressed as the mean values ± standard deviations (SDs) derived from triplicate samples. Cluster analysis was conducted using heatmap tools available at Hiplot Pro (https://hiplot.com.cn/, accessed on 30 May 2023), a comprehensive web service for analyzing and visualizing biomedical data, to classify the antimicrobial activities of the LAB isolates against pathogens.

## 3. Results

### 3.1. Isolation of LAB Strains

**Figure 1:**
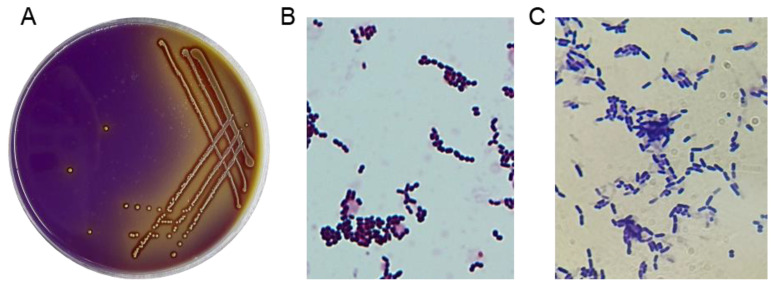
Isolation of LAB strains. (**A**) LAB strains were visually screened using MRS agar plates with bromocresol blue and CaCO_3_. (**B**,**C**) The results of the gram staining test on the suspected LAB isolates (1000×).

A total of 44 potential LAB strains were isolated from the intestinal tracts of healthy broilers based on the distinct calcium-dissolving zone and discoloration reaction (from purple to yellow) around the colonies on MRS screening plates (Figure 1A). All 44 isolates were catalase-negative and Gram-positive, displaying typical morphological features such as creamy-white and fruity colonies and coccus- or rod-shaped characteristics (Figure 1B,C), which enabled their morphological classification as LAB.

### 3.2. Antimicrobial Activity In Vitro

**Figure 2:**
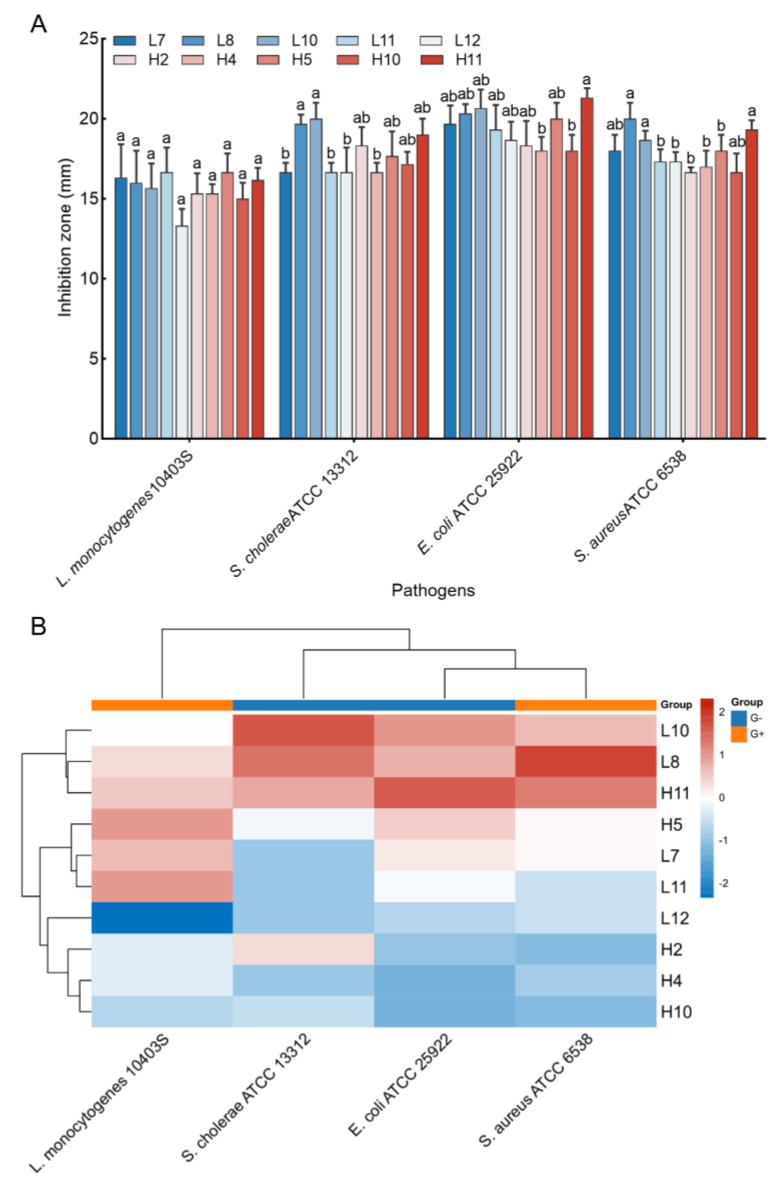
Antimicrobial activity of the LAB strains against pathogens. (**A**) Inhibition zones of the CFS of the strains against four pathogen indicators. The results represent the mean ± SD of three replicates. For each pathogen indicator, different letters on each bar represent significant differences between values (*p* < 0.05). (**B**) Cluster analysis was undertaken to comprehensively assess the antimicrobial activities of LAB isolates against pathogens by using heat map tools in Hiplot Pro (https://hiplot.com.cn/, accessed on 30 May 2023).

Antimicrobial activity against four pathogen indicators (*S*. *aureus* ATCC 6538, *L*. *monocytogenes* ATCC 10403S, *E*. *coli* ATCC 25922, and *S*. *cholerae* ATCC 13312) was assessed for the 44 LAB isolates. Results indicated that only 10 LAB isolates displayed significant inhibition activities against all tested pathogens, with inhibition zones ranging from 13.33 ± 1.04 to 21.33 ± 0.58 mm (Figure 2A). Notably, H5 and L11 exhibited the largest inhibition zone against *L*. *monocytogenes* ATCC 10403S (16.67 mm), while L8 and L10 yielded the most significant inhibition zone against *S*. *cholerae* ATCC 13312 (19.67 and 20.00 mm, respectively, *p* > 0.05). H11 exhibited the largest inhibition zone against *E*. *coli* ATCC 25922 (21.33 mm), and L8, H11, and L10 yielded larger inhibition zones against *S*. *aureus* ATCC 6538 (20.00, 19.33, and 18.67 mm, respectively, *p* > 0.05). Cluster analysis using the heatmap method demonstrated that three LAB strains (L10, L8, and H11) exhibited favorable antimicrobial activity against all tested pathogens (Figure 2B), leading to their selection for further analyses.

### 3.3. Characterization of LAB Antimicrobial Compounds Produced by Strains

The selected LAB strains underwent characterization to produce antimicrobial compounds, including organic acids, hydrogen peroxide, and bacteriocins. The CFS of LAB strains, both before and after catalase treatment, exhibited nearly the same inhibitory activity against all tested pathogens, suggesting that the inhibition was not attributed to the production of hydrogen peroxides. However, the inhibitory effect of CFS after protease K treatment was significantly reduced, especially for strains H11 and L10, implying that the antimicrobial compounds are proteinaceous. Heat treatment did not affect the inhibitory activities of the CFS of LAB strains tested, indicating that the antimicrobial compounds produced by these strains were heat-stable. Furthermore, when the pH of the CFS of different LAB cultures was increased (pH 5.0), the antimicrobial activity dramatically decreased compared to the untreated CFS (Table 1). This effect disappeared when the CFS was neutralized at pH 5.5, suggesting that acid production significantly contributed to the inhibitory effect of these LAB strains, including the action of bacteriocins.

### 3.4. Acid and Bile Tolerance Ability of LAB Strains

**Figure 3:**
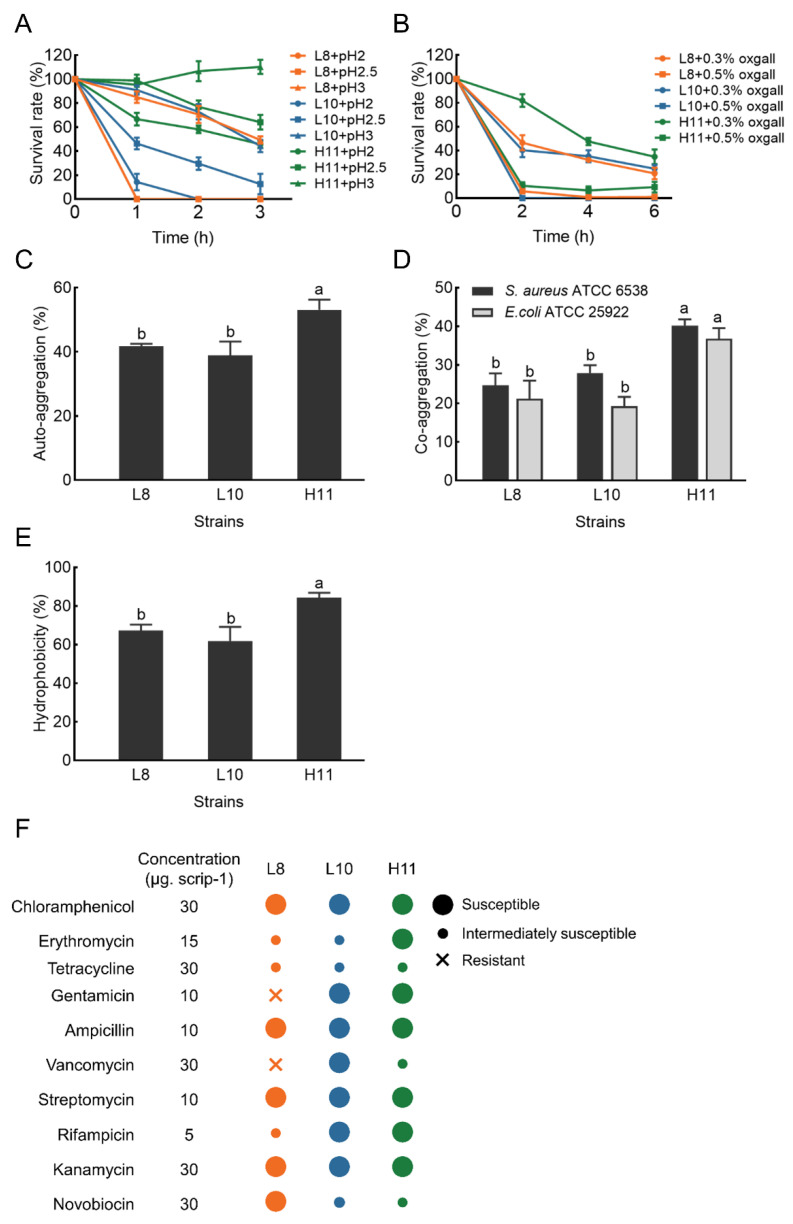
The probiotic characteristics of strains L8, L10 and H11. (**A**) Acid tolerance; (**B**) bile tolerance; (**C**) Auto−aggregation; (**D**) Co−aggregation; (**E**) Hydrophobicity; (**F**) Antibiotic resistance dashboard. The results represent the mean ± SD of three replicates. Different letters on each bar represent significant differences between values (*p* < 0.05).

The acid tolerance ability of the three selected isolates is shown in Figure 3A. H11 yielded the highest survival rate after 3 h of incubation in an acidic environment (pH 2.0–3.0), with values ranging from 45% to 110%. L8 and L10 demonstrated moderate ability to survive at pH 3.0 after 3 h of incubation (49% and 44%, respectively). However, the survival rate of L8 and L10 was very low at pH < 3.0, particularly at pH 2.0.

Simultaneously, H11 exhibited excellent tolerance in the presence of 0.3% bile salt and maintained a survival rate ranging from 35% to 81% after 2.0–6.0 h of incubation. Under the same conditions, L8 and L10 exhibited moderate bile salt tolerance, with a survival rate of 24−46%. However, the survival rate of the three LAB strains was less than 10% in the presence of 0.5% bile salt (Figure 3B).

### 3.5. Cell Surface Characteristics

#### 3.5.1. Auto-Aggregation and Co-Aggregation Ability

Auto-aggregation results of the selected isolates are displayed in Figure 3C. The auto−aggregation of these isolates ranged from 38.90 ± 4.31 to 53.12 ± 3.14%. After 4 h of incubation, the auto-aggregation of strain H11 was significantly higher (*p* < 0.05) than strains L8 and L10.

Co−aggregation results of these LAB strains with two pathogens (*E. coli* ATCC 25922 and *S. aureus* ATCC 6538) are shown in Figure 3D. All three LAB strains exhibited stronger co−aggregation ability with *S. aureus* ATCC 6538 compared to *E. coli* ATCC 25922. The co−aggregation of strain H11 with pathogens was 40.20% (*S. aureus* ATCC 6538) and 36.81% (*E. coli* ATCC 25922), respectively. H11 demonstrated significantly higher co−aggregation ability with the two pathogens than L8 and L10 (*p* < 0.05).

#### 3.5.2. Cell Surface Hydrophobicity

The cell surface hydrophobicity results of the three LAB strains are presented in Figure 3E. The hydrophobicity value of strain H11 was 84.31% in xylene, significantly higher than that of L8 (67.20%) and L10 (61.81%) (*p* < 0.05).

### 3.6. Safety Evaluation In Vitro of LAB Strains

The hemolytic activity of the three strains was examined based on the presence of hemolytic halos around the colonies on blood agar plates. None of the three strains exhibited a zone of hemolysis or a zone of greenish to brownish discoloration, indicative of γ−hemolysis (non−hemolytic activity). Figure 3F demonstrates the susceptibility profile of the three LAB isolates to several commonly used antibiotics, confirming their susceptibility to most antibiotics. Specifically, all three LAB isolates were susceptible to chloramphenicol, penicillin, ampicillin, streptomycin, and kanamycin. Two (L10 and H11) were susceptible to gentamicin and rifampicin, while one was susceptible to erythromycin (H11), vancomycin (L10), and novobiocin (L8). Nevertheless, strain L8 was resistant to gentamicin and vancomycin.

### 3.7. Molecular Identification of LAB Strains

**Figure 4:**
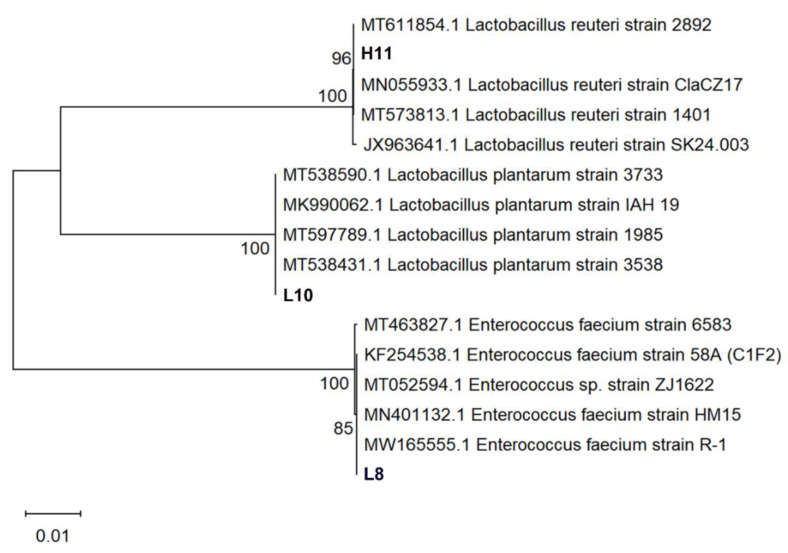
Phylogenetic analysis of strains L8, L10, and H11 based on 16S rRNA gene sequences. The isolates sequenced in the study are depicted in bold font. The tree was constructed with the neighbor-joining method by using MEGA 11. The percentage of replicate trees in which the associated taxa clustered together in the bootstrap test (1000 replicates) are shown next to the branches. The evolutionary distances were computed using the Kimura two-parameter method and are in the units of the number of base substitutions per site.

Three LAB strains were identified through 16S rRNA sequencing. Phylogenetic analyses of 16S rDNA sequences of the isolates revealed that strain L8 clustered into the clade of *Enterococcus faecium* (*E. faecium*), indicating a genetic homology of 99.72% with *E*. *faecium* strain HM15 (Accession No.:MN401132). Isolate L10 belonged to the cluster of *Lactiplantibacillus plantarum* (*L*. *plantarum*) with 99.66% genetic homology to *L*. *plantarum* IAH 19 (Accession No.: MK990062). Strain H11 was classified in the cluster of *Limosilactobacillus reuteri* (*L*. *reuteri*) with 99.59% genetic homology to *L*. *reuteri* strain ClaCZ17 (Accession No.: MN055933) isolated from chickens (Figure 4). The three LAB isolates were identified as *E. faecium* L8, *L*. *plantarum* L10, and *L*. *reuteri* H11. These lactobacilli are registered on the GenBank website (https://www.ncbi.nlm.nih.gov/genbank/, accessed on 30 November 2023.) with the accession numbers: OR879323 (L8), OR879324 (H11), and OR879325 (L10).

## 4. Discussion

In recent years, probiotics have attracted significant interest and are now considered a promising alternative to the widespread use of antibiotics in the poultry industry. Most probiotics are sourced from the microbial communities found in the intestines of animals and humans and in dairy products [8]. Typically, probiotic strains derived from their natural hosts are preferred over strains from other sources because of their increased host adaptation, higher viability, greater safety, and more effective delivery of probiotic effects [19]. Moreover, since the antagonistic effect of probiotics against pathogens is the main influential factor that hinders the colonization of heterochthonous bacteria in the GI tract, the use of indigenous microorganisms as probiotics is expected to make an important contribution to the control of pathogens [27]. Similarly, Jose et al. [28] stated that LAB strains from animal rumen yielded a better inhibitory effect on pathogen growth than those from dairy products. Therefore, developing host-specific probiotics is essential to facilitate animal health and optimal performance [20]. To this end, LAB strains were selectively isolated from healthy broilers, and their potential for development as poultry probiotics was evaluated in this work. Bromocresol purple and CaCO_3_ (as pH indicators) were supplemented into the MRS screening plates for visually selecting LAB strains. The emergence of a yellow area and a calcium-dissolving zone around a bacterial colony on the MRS screening plates indicated that the bacterium could produce acid or make the environment acidic [29]. After isolating 44 strains of LAB from the intestinal tracts of broilers, they were subjected to in vitro evaluation of probiotic properties and final molecular characterization.

It is widely acknowledged that infections caused by zoonotic and food−borne enteric pathogens can lead to high morbidity and mortality, ultimately resulting in significant economic losses in the poultry industry [30]. Thus, antimicrobial activity against these pathogens is a major requirement for selecting potential probiotics [5]. In this study, 10 of 44 LAB strains showed broad-spectrum antimicrobial activity against the four pathogens tested (Figure 2A). Moreover, these LAB strains had comparable antimicrobial activity against Gram−negative and Gram-positive bacteria, but the least-inhibitory zones were observed against *Listeria monocytogenes*. Our findings are similar to many previous studies that reported antimicrobial activity by LAB strains sourced from poultry against broad-spectrum pathogens [5,11,19,31]. Conversely, some researchers revealed that LABs isolated from the chicken gastrointestinal tract have high inhibitory activity against Gram-positive pathogens (*Clostridium perfringens*) but low inhibitory activity against Gram-negative pathogens (including *Escherichia coli* and *Salmonella enteritidis*) [32]. However, when the inhibitory activity of LAB is due to lactic acid, acetic acid, or hydrogen peroxide, it was independent of the Gram type of pathogens tested [33]. Admittedly, the antagonistic activity of probiotics against a broad spectrum of pathogens is a significant property, as it can provide valuable prospects for their use as feed additives, or in veterinary medicine [34]. Therefore, out of the LAB strains examined, three were selected as candidate probiotics for subsequent evaluation, given their favorable antimicrobial activities against the tested pathogens.

The antimicrobial activity of LAB is maintained by the secretion of different antibacterial components, such as hydrogen peroxide, organic acids (lactic and acetic acids), bacteriocins, alcohols, diacetyl, peptides, etc., which prevent or reduce the growth of pathogenic bacteria in the gut [30,35,36,37]. Our findings revealed that the antimicrobial activity of the LAB strains tested was not a result of hydrogen peroxide production, as it was almost the same in the untreated and catalase−treated supernatants of LAB strains. Many LAB strains can produce bacteriocins or bacteriocin-like components—a class of small molecules, cationic, hydrophobic, and heat−stable peptides [38]. LAB-derived bacteriocins have potent antibacterial properties and strong bactericidal or bacteriostatic effects against various pathogens through specific mechanisms, such as targeting bacterial membrane integrity and septum formation during mitosis [39]. Bacteriocin production has always been considered an important parameter in selecting probiotics [40]. The secretion of bacteriocins can promote the probiotic activity of intestinal LAB and, in some cases, may be directly responsible for beneficially regulating the intestinal microflora or inhibiting some pathogens [41]. Therefore, bacteriocins and bacteriocinogenic LAB are considered excellent alternatives to antibiotics [42] and have been widely used in the food industry, medicine, veterinary medicine, animal feed, and other fields [43,44]. So far, increasing numbers of bacteriocinogenic LAB strains have been isolated and characterized. For example, Miao et al. [45] characterized a novel bacteriocin F1 secreted by *L. paracasei* strain FX-6 isolated from Tibetan kefir. Our findings showed that the antimicrobial components of the selected LAB, especially strains H11 and L10, contained heat-stable substances sensitive to proteases, such as bacteriocins, suggesting that the selected LAB strains have huge prospects as potential probiotics. LAB can also produce a variety of organic acids in different concentrations, and lactic acid is usually one of its major metabolites [46]. Indeed, many LAB strains exert antimicrobial activity largely due to the secretion of lactic acid [47]. In addition, the antibacterial effects of other acids, such as citric acid and tartaric acid, have been reported previously [48]. The antimicrobial mechanisms of organic acids vary widely, affecting cell function and development by acidifying the cytoplasm and inhibiting the activity of acid-sensitive enzymes [49]. In the present study, the antimicrobial properties of the selected LAB strains were significantly inhibited in an acidic environment at pH 5.0, showing only weak to moderate inhibitory activity, which disappeared completely at pH 7.0. The above results indicate that the selected LAB strains contained antimicrobial substances other than organic acids and that these substances were acid-dependent, exerting antimicrobial activity only in an acidic environment. Our findings are consistent with those of Niku-Paavola et al. [50], who indicated that inhibitory substances produced by *L. plantarum* E76 were active only at low pH and in the presence of lactic acid. In addition, Makras et al. [51] reported that the antagonistic effect of the antimicrobial compounds produced by *L. johnsonii* La1 and *L. plantarum* ACA-DC 287 was significant only in the acidic environment of lactic acid, which may be due to lactic acid acting as a permeabilizer of the bacterial outer membrane, which may trigger the antimicrobial activity of other inhibitory compounds when a certain concentration is reached [52]. In future work, it is necessary to further characterize the antimicrobial components of the selected LAB and explore the molecular mechanisms underlying their antimicrobial properties.

The ability of potential probiotics to withstand various adverse factors in the gastrointestinal tract (digestive enzymes, bile salts, low pH gastric acid, etc.) is crucial to their colonization and proliferation in the host gut. Therefore, this is the major requirement for screening and evaluating probiotics [53,54]. According to previous studies, pH 2.0–3.0 [55,56] and 0.3% bile salts [11,57] were used as thresholds of acid and bile salt tolerance tests for potential probiotics, respectively. *L. plantarum* strain OF101, isolated from a fermented cereal beverage, showed 98.4% and 96.9% survival rates at pH 2.5 and 0.3% bile salts, respectively [58]. Jang et al. [23] reported that the survival rate of *L. brevis* KU15153, isolated from kimchi, was 52.48% and 101.91% under artificial gastric conditions (pH 2.5, 0.3% pepsin for 3 h) and bile salt conditions (0.3% bile salts for 24 h), respectively. In addition, MA2 showed a survival of 70% at pH 2.5 and 0.3% bile salt for 3 h [59]. In another study, six LAB strains isolated from chickens showed good tolerance to 0.3% bile salt after 6 h of exposure and moderate-to-good survival in simulated gastric juice with a pH of 2.0 [19]. In comparison, our isolate, H11, was found to possess moderate-to-good survivability under pH 2.0–3.0 and 0.3% bile salt conditions, indicating that it could be considered a promising candidate for use as a probiotic.

The hydrophobicity and aggregation abilities of probiotics correlate strongly with their adhesion to gastrointestinal epithelial cells. Therefore, these are two important characteristics for evaluating and selecting potential LAB probiotics [31]. The hydrophobicity of probiotics’ cell surface gauges their capacity to adhere to enterocyte cell lines [60], a highly desirable characteristic in probiotics. Some LAB strains have shown a correlation between hydrophobicity and adhesion [53]. In general, probiotics with high hydrophobicity values exhibited a greater ability to adhere to epithelial cells [61]. García-Hernández et al. [11] reported a hydrophobicity of 71.10% for *L. pentosus* LB-31 isolated from broiler excreta. In this work, all three LAB strains tested showed high hydrophobicity, ranging from 61.81% to 84.31%, indicating their strong adhesion to epithelial cells and mucosal surfaces. In two other studies, the hydrophobicity of LAB strains isolated from poultry ranged between 40.5–71.0% [19] and 21.18–95.27% [22]. Such discrepancy among the LAB strains tested may be attributed to differences in the hydrophobic moieties of surface proteins in the cell wall [62]. Significantly, a moderate level of hydrophobicity does not necessarily mean a lower adhesion ability of the bacteria, as hydrophilic domains may also engage in bacterial adhesion [63]. The aggregation ability of bacteria has usually been connected with adhesion properties [64]. Auto-aggregation facilitates probiotics to adhere to and colonize host intestinal cells, which is critical in several ecological niches [6,30]. According to Roghmann and McGrail [65], auto-aggregation above 40% is necessary for a strain to be a potential probiotic. In the current study, the auto−aggregation of the selected LAB strains was between 38.90 ± 4.31% and 53.12 ± 3.14%, indicating their acceptable adhesion ability. Our findings are consistent with a study by Reuben et al., who reported that the auto−aggregation of LAB strains isolated from poultry ranged from 32 ± 5.66% to 56.5 ± 3.54% [19]. Co-aggregation measures the adherence of tested strains to the enteric pathogens [6]. Therefore, the co-aggregation capacity of probiotic strains may make it possible to form a barrier that effectively hinders the adhesion and colonization of enteric pathogens on intestinal cells [30]. Chandran and Keerthi reported 63.66% co-aggregation for *L. plantarum* MBTU−HK1 with *S. typhi* [66]. In this work, the co-aggregation abilities between the three LAB strains and the pathogens were significantly different, and the co−aggregation value ranged between 21.21 and 36.81% for *E. coli* and 24.71 and 40.20% for *S. aureus*. In another investigation, Sophatha et al. [67] also reported that the co-aggregation value ranged between 21 and 32% for different LAB strains with *E. coli*. In addition, the co-aggregation abilities between the three LAB strains tested and pathogens were strain- and pathogen-specific, and the co-aggregation value of LAB strains and *S. aureus* was higher than that of *E. coli*. Findings from the work of Reuben et al. [19] also reported strain- and pathogen-specific co-aggregation abilities between the potential LAB probiotics and the six pathogens.

Probiotic candidates should not serve as reservoirs for antibiotic resistance genes [68], as such genes may further be transferred to enteric pathogens. Therefore, assessing the antimicrobial susceptibility profile is considered essential for selecting and safely using potential probiotic strains [69]. All three LAB isolates were susceptible to chloramphenicol, penicillin, ampicillin, streptomycin, and kanamycin; two were susceptible to gentamicin and rifampicin, while one was susceptible to erythromycin, vancomycin, and novobiocin. It has been reported that *lactobacilli* are generally susceptible to ampicillin [70]. In a recent study, Reuben et al. reported high susceptibility to chloramphenicol, penicillin, ampicillin, and novobiocin in LAB strains isolated from poultry [19]. Likewise, Dowarah et al. [20] mentioned that LAB strains isolated from pigs and poultry were highly susceptible to penicillin, ampicillin, and chloramphenicol. Interestingly, only one strain of LAB in this study was resistant to gentamycin and vancomycin. It has been documented that LAB is intrinsically resistant to gentamicin and vancomycin due to their impermeable membranes [28]. Meanwhile, the absence of hemolytic activity has been recommended as a safety characteristic for selecting probiotics [54]. Our results revealed that the three LAB strains showed non-hemolytic activities, depicting their non-virulent nature and safety in vivo. Similar findings showed that most LAB strains were non-hemolytic, as previously reported [6]. Building upon their antibiotic sensitivity profile and hemolytic activity in vitro, the three LAB strains could meet the basic requirements of probiotics, which makes them safe candidates for further verification in future probiotic studies in vivo.

## 5. Conclusions

Among the three LAB isolates from poultry, *Limosilactobacillus reuteri* H11 showed favorable in vitro probiotic characteristics, including broad-spectrum antimicrobial activity against pathogens, high cell surface properties, good acid (pH 2.0–3.0) and bile salt tolerance, and no antibiotic resistance or hemolytic activity. Therefore, *L. reuteri* H11 may be a great candidate for probiotics, which can be used as a poultry feed supplement to promote animal health and production performance. Of course, in vivo animal tests and whole−genome analysis are recommended to confirm the application potential of this strain.

## Figures and Tables

**Table 1:** Characterization of antimicrobial compounds from three LAB isolates.

Treatment	Residual Inhibitory Activity (mm) ^#^					
	*E. coli* ATCC 25922			*S. aureus* ATCC 6538		
	L8	L10	H11	L8	L10	H11
Untreated (pH 3.0)	21.00 ± 1.00	20.33 ± 0.76	21.67 ± 0.76	19.67 ± 1.15	19.00 ± 0.00	19.33 ± 0.58
pH 5.0	10.33 ± 0.58 *	10.33 ± 1.04 *	16.00 ± 1.73 *	11.33 ± 0.58 *	10.67 ± 1.15 *	13.33 ± 0.76 *
Catalase	19.67 ± 1.04	19.50 ± 0.87	20.33 ± 1.26	18.00 ± 0.87	18.33 ± 0.29	18.67 ± 1.04
Proteinase K	18.83 ± 1.53	16.67 ± 0.58 *	15.67 ± 0.29 *	15.17 ± 0.76 *	14.33 ± 0.58 *	13.33 ± 0.76 *
Heat (100 °C, 20 min)	20.67 ± 0.76	20.00 ± 0.87	20.50 ± 0.50	19.17 ± 0.76	18.00 ± 0.87	19.17 ± 1.04

^#^ Inhibition halo (mm). * represents a significant difference (*p* < 0.05) in means between the treatment and control groups.

## Data Availability

The data underlying this article will be shared upon reasonable request to the corresponding author.
